# Depression and Crime Across Different Neighborhoods in the Swedish General Population

**DOI:** 10.1001/jamanetworkopen.2025.57546

**Published:** 2026-02-03

**Authors:** Nilo Tayebi, Anneli Andersson, Seena Fazel, Henrik Larsson, Brittany Evans, Catherine Tuvblad

**Affiliations:** 1 School of Behavioural, Social, and Legal Sciences, Örebro University, Örebro, Sweden; 2 Department of Psychiatry, University of Oxford, Oxford, United Kingdom; 3 Oxford Health NHS Foundation Trust, Oxford, United Kingdom; 4 School of Medical Sciences, Örebro University, Örebro, Sweden

## Abstract

**Importance:**

Depression and factors reflecting neighborhood social structure (ie, socioeconomic deprivation, ethnic heterogeneity, residential mobility, and urbanicity) have each been linked to criminal convictions. However, how the association between depression and crime varies across different neighborhood types, and the extent to which it reflects unmeasured familial confounding, remains unclear.

**Objective:**

To examine whether the association between depression and violent and nonviolent criminal convictions varies across neighborhood types, and to assess the extent to which unmeasured familial factors contribute to the association.

**Design, Setting, and Participants:**

This population-based matched cohort and sibling-comparison study used data from Swedish national registers from 1986 to 2020. Follow-up spanned from 2001 to 2020. Statistical analyses were performed from January to November 2025. The cohort included individuals with a diagnosis of depression, each matched to 5 population controls without depression by birth year, sex, and neighborhood type.

**Exposure:**

Outpatient depression diagnosis (*International Statistical Classification of Diseases and Related Health Problems, Tenth Revision* codes F32-F33.9) recorded from 2001 to 2020.

**Main Outcomes and Measures:**

The primary outcomes were violent and nonviolent criminal convictions after diagnosis, identified through the National Crime Register. Conditional logistic regression estimated odds ratios (ORs) across 4 neighborhood types (resource-limited, rural low-diversity, urban professional, and urban affluent neighborhoods), with sibling comparisons used to assess familial confounding.

**Results:**

Among 571 470 matched individuals, 95 245 (36 297 male [38.1%]; median [IQR] age at first diagnosis, 20 [17-24] years) had depression. Depression was associated with increased odds of both violent and nonviolent convictions across all neighborhood types in unadjusted models. After adjustment for prior convictions, substance use disorder, and attention-deficit/hyperactivity disorder, associations remained significant in all but resource-limited neighborhoods (violent conviction OR, 1.14 [95% CI, 0.97-1.33]; nonviolent conviction OR, 1.01 [95% CI, 0.92-1.11]). A second sample included 42 585 individuals with depression and their full siblings without depression (total, 85 170 individuals). Sibling comparisons showed partial attenuation, indicating that familial confounding accounted for some, but not all, of the associations. Sibling-matched estimates were largely consistent with fully adjusted general population-matched estimates (eg, violent convictions in rural low-diversity neighborhoods: sibling-matched OR, 1.50 [95% CI, 1.33-1.69] vs general population-matched OR, 1.51 [95% CI, 1.39-1.65]).

**Conclusions and Relevance:**

In this cohort study of the Swedish general population, the association between depression and criminal convictions varied across neighborhood types and was partially explained by familial factors. These findings underscore the relevance of considering contextual and familial influences and may offer insights for prevention and intervention strategies responsive to neighborhood social environments.

## Key Points

**Question:**

Does the association between depression and violent and nonviolent crime differ across neighborhoods, and do unmeasured familial factors contribute to it?

**Findings:**

In this cohort study of 95 245 individuals with depression and 476 225 controls, depression was significantly associated with higher odds of both violent convictions and nonviolent convictions. Associations were lowest in resource-limited neighborhoods and were partially attenuated in sibling analyses.

**Meaning:**

These findings suggest that depression is associated with increased risk of criminal convictions across neighborhood types and underscore the relevance of considering contextual and familial factors for prevention and intervention strategies responsive to neighborhood social environments.

## Teaser

This cohort study examines whether the association between depression and violent and nonviolent criminal convictions varies across neighborhood types and assesses the extent to which unmeasured familial factors contribute to the association.

## Introduction

The link between depression and crime has received growing attention, with evidence suggesting that individuals with depression face increased risk of violent and nonviolent convictions.^1,2,3,4,5^ The nature of this association remains poorly understood. Individual-level factors such as poor impulse control, affect regulation, and low self-control have been suggested as potential mechanisms.^6,7^ Beyond individual factors, environmental factors, particularly neighborhood social structure, may shape the social conditions and stressors contributing to both mental health and criminal behavior.^8,9,10^

Neighborhood social structure includes factors such as socioeconomic deprivation, ethnic heterogeneity, residential mobility, and urbanicity.^10^ General Strain Theory suggests that psychological distress, including depression, can increase the risk of criminal offending, and that this risk is shaped by broader social environments like neighborhood social structure.^11,12^ Social Disorganization Theory posits that structural disadvantages weaken cohesion and informal control, fostering crime.^10,13,14^ Economically disadvantaged areas often lack key resources,^15,16^ increasing crime risk.^17,18^ Ethnic heterogeneity and residential mobility can hinder communication^13^ and stable relationships,^19^ further elevating crime risk.^20,21,22^ Neighborhood social structure is also linked to depression.^23^ Specifically, urban living, socioeconomic deprivation, and high residential mobility have been linked to an increased depression risk.^24,25,26,27^ Taken together, these perspectives suggest that the link between depression and crime may be amplified in disadvantaged, socially disorganized neighborhoods and attenuated in resource-rich, cohesive ones.

However, some research suggests that the association between depression and crime may be partially confounded by unmeasured familial factors—that is, heritable and shared environmental influences (eg, parental mental illness, criminality, or low socioeconomic status) that are associated with the risk of both depression and crime.^2,11,28^ Depression and criminal behavior aggregate in families, suggesting heritable components.^29,30^ This association may also vary by neighborhood characteristics: heritable contributions to criminal behavior appear stronger in higher socioeconomic neighborhoods,^31^ whereas heritable contributions to depression have been found to increase with neighborhood deprivation.^32^ Thus, these findings point to both substantial familial contributions and heterogeneity in the associations.

Although most studies on depression and crime focus on violent crime,^2,5^ less is known about nonviolent crime, which may show different associations with depression^1,4^ and differing familial effects.^33,34^ Also, prior research often examined neighborhood factors (eg, socioeconomic deprivation) in isolation, modified by or while controlling for other neighborhood characteristics (eg, urbanicity).^35^ Although such research is etiologically valuable, it limits ecological validity and social applicability, as neighborhood structure involves multiple interrelated factors. We applied latent profile analyses (LPAs) to Swedish population-based data^36^ to identify empirically derived neighborhood types that reflect this complexity. This multidimensional approach aligns with theoretical perspectives that view neighborhood social structure as an emergent property of interrelated factors and may offer greater ecological validity and social applicability. It allows us to capture the kinds of composite neighborhoods in which residents live, and policy interventions are typically implemented. Although depression and neighborhood characteristics may independently be associated with increased crime risk, it remains unclear whether their association varies across neighborhood types. Clarifying the extent of familial confounding is key to understanding causality, as prior studies suggest shared familial factors may partly explain the association between depression and crime^2,11,28^ with possible variation by neighborhood characteristics.^31,32^

The present study addressed 2 research questions. First, how does the association between depression and subsequent violent and nonviolent criminal convictions vary across different neighborhood types? Second, to what extent do unmeasured familial factors contribute to the associations between depression and subsequent violent and nonviolent criminal convictions within each neighborhood type?

On the basis of prior research,^4,17,25^ we hypothesized that depression would be associated with increased likelihood of subsequent violent and nonviolent convictions, with greater associations in disadvantaged neighborhoods. We also hypothesized unmeasured familial confounding to partly explain these associations within each neighborhood type, on the basis of previous studies.^2,11,28^ Neighborhood types were based on prior LPAs of Swedish population-based data.^36^

## Methods

This study was approved by the Swedish Ethical Review Authority. Under Swedish law, informed consent is not required for research using pseudo-anonymized register data, provided that such use has been approved by an ethics review board.^37^ The study was conducted in accordance with the Strengthening the Reporting of Observational Studies in Epidemiology (STROBE) reporting guidelines for cohort studies.^38^

### Data Sources

The following registries were used to identify the study cohort: Total Population Register (TPR),^39^ National Patient Register (NPR),^40,41^ National Crime Register (NCR), Multi-Generation Register (MGR),^42^ Longitudinal Integration Database for Health Insurance and Labor Market Studies (LISA),^43^ and the Demographic Statistical Areas (DeSO).^44^ Details are available in the eMethods in Supplement 1.

### Study Population

Using the TPR, we identified all individuals born between 1986 and 2005 (2 875 441 individuals). Exclusions were based on migration, early mortality, psychiatric diagnoses (eg, schizophrenia spectrum or bipolar disorders), and missing neighborhood classification (see eMethods in Supplement 1). To mitigate the risk of reverse causation, individuals with inpatient depression episodes were excluded. The final cohort comprised 1 850 142 individuals (52% male). As outpatient data in the NPR became available in 2001,^40,41^ follow-up began on January 1, 2001, or when individuals turned 15 years, the minimum age of criminal responsibility in Sweden, and ended December 31, 2020.

### Exposure and Outcomes

Individuals with a diagnosis of depression were identified in the NPR according to at least 1 outpatient *International Statistical Classification of Diseases and Related Health Problems, Tenth Revision (ICD-10)* diagnosis (codes F32-F33.9) between 2001 and 2020. Criminal convictions were obtained from the NCR. Only convictions following the depression diagnosis were treated as outcomes; earlier convictions were treated as a covariate (prior convictions). We examined 2 separate outcomes: convictions for violent and nonviolent crimes. Following established criteria,^29^ violent criminal convictions included convictions for homicide, manslaughter, assault, kidnapping, illegal confinement, unlawful coercion, gross violation of a person’s integrity, unlawful threats, intimidation, robbery, arson, and threats or violence against an officer. Sexual offenses, including rape, sexual coercion, molestation, and sexual harassment, were included in this category. Nonviolent criminal convictions included all other convictions (eg, theft and fraud).

### Covariates

We adjusted for prior convictions,^45,46^ defined as any conviction before the first outpatient diagnosis of depression, using NCR data. We also adjusted for comorbid substance use disorders (SUD) and attention-deficit/hyperactivity disorder (ADHD), given their associations with depression and crime.^47,48,49,50,51^ Diagnoses were identified through inpatient or outpatient records in the NPR using the following codes: SUD (*International Classification of Diseases, Eighth Revision* codes 303 and 304; *International Classification of Diseases, Ninth Revision [ICD-9]* codes 303, 304, 305.1, and 305.9;* ICD-10* codes F10-F19) and ADHD (*ICD-9* code 314; *ICD-10* code F90).

### Neighborhood Social Structure

Neighborhood social structure was classified using LPAs,^36^ on the basis of 6234 distinct DeSO neighborhoods and indicators from Swedish national registers (DeSO, TPR, LISA, and MGR). These indicators captured socioeconomic conditions, ethnic heterogeneity, residential instability, and urbanicity. Five neighborhood types were identified: rural resource-limited, urban high-diversity, rural low-diversity, urban professional, and urban affluent neighborhoods (Table 1).

**Table 1.** Description of Neighborhood Types Previously Identified Through Latent Profile Analyses of Swedish Population-Based Data

Neighborhood type	Neighborhoods, No. (% (N = 6234))^a^	Description
Rural resource-limited neighborhoods	222 (3)	Lowest scores on education and income; highest scores on residential instability, social benefit recipients, and proportion of unmarried individuals; and the second-highest score on ethnic heterogeneity
Urban high-diversity neighborhoods	559 (9)	Highest scores on ethnic heterogeneity and the second-lowest scores on income, education, and social benefit recipients
Rural low-diversity neighborhoods	2847 (46)	Lowest scores on ethnic heterogeneity and urbanicity, as well as low scores on residential instability
Urban professional neighborhoods	1188 (19)	Highest scores on urbanicity, and the second-highest scores on residential instability, proportion of unmarried individuals, and education
Urban affluent neighborhoods	1418 (23)	Highest scores on income and education, and the lowest scores on residential instability, proportion of unmarried individuals, and social benefits recipients

^a^ Frequencies reflect the distribution of Demographic Statistical Areas neighborhoods in 2001 and 2005.^36^ Demographic Statistical Areas neighborhood counts within each neighborhood type varied modestly across time points, but proportional distributions remained consistent.

Owing to limited sample sizes, the 2 smallest and most similar types, rural resource-limited and urban high-diversity, were combined into a single resource-limited neighborhood type. This type had the lowest income and education, highest ethnic heterogeneity and proportion of social benefit recipients, mid-to-high residential instability, a higher proportion of unmarried individuals, and moderate urbanicity (see eMethods in Supplement 1).

### Matching Procedure

#### General Population–Matched Sample

SAS statistical software version 9.4 (SAS Institute) was used to construct 2 matched samples, comprising a general population–matched sample and a sibling-matched sample. Each individual with an outpatient depression diagnosis (case) was matched to 5 unique controls without a depression diagnosis from the general population by birth year period, sex, and neighborhood type. To ensure an equal distribution of controls for each case, and to maximize the available number of controls, 5 unique controls were selected per case through a random selection process.

#### Sibling-Matched Sample

Individuals with an outpatient depression diagnosis (cases) were matched 1:1 to their full siblings without a depression diagnosis (controls), identified through MGR and restricted to siblings sharing the same neighborhood type. In sibling groups with unequal numbers of siblings with and without depression, random selection was applied to maintain the 1:1 matching ratio.

For both samples, follow-up began at the first outpatient depression diagnosis, ensuring a uniform baseline between cases and controls. Convictions prior to diagnosis were treated as prior convictions (covariate), and later convictions were classified as violent or nonviolent criminal conviction outcomes. Full matching details are available in the eMethods and eFigures 1 and 2 in Supplement 1.

### Statistical Analysis

All data management and statistical analyses were performed using SAS software version 9.4 (SAS Institute) and RStudio version 2023.09.0 (R Project for Statistical Computing). Statistical analyses were performed from January to November 2025. First, to assess whether the association between depression and criminal convictions varied across distinct neighborhood types, we conducted conditional logistic regression models on the general population–matched sample, estimating odds ratios (ORs) and 95% CIs. Analyses were stratified by neighborhood type and conducted separately for violent and nonviolent criminal convictions. Covariates were adjusted stepwise: (1) unadjusted, (2) adjusted for prior convictions, (3) additionally adjusted for SUD, and (4) additionally adjusted for ADHD. Wald tests assessed differences across neighborhood types. Comparisons were first made using unadjusted models to assess differences prior to covariate adjustment and then repeated on fully adjusted models. Rural low-diversity neighborhoods served as the reference group because of their largest representation (2847 neighborhoods [46%]), and Bonferroni correction accounted for multiple testing.

Second, to assess unmeasured familial confounding, we applied conditional logistic regression models to the sibling-matched sample to estimate ORs and 95% CIs, conditioning on full sibling pairs. This approach compares siblings discordant for depression while holding constant shared familial factors (eg, genetics and early environment). Analyses were stratified by neighborhood type and were conducted separately for violent and nonviolent criminal convictions. Sibling estimates were compared with fully adjusted population-matched estimates using Wald tests. The comparison of discordant sibling pairs helps assess whether shared familial factors confound the association between depression and criminal convictions. If such confounding is present, we would expect attenuated ORs in the sibling comparison compared with the general population–matched estimates.

We conducted a sensitivity analysis further stratifying the general population–matched sample by SUD. Given associations between substance-related problems and both depression and crime,^49,50^ as well as substance-related problems and neighborhood social structure,^52,53^ this analysis aimed to clarify the role of SUD by examining whether the association between depression and criminal convictions across neighborhood types persisted irrespective of comorbidity. In addition, because the exposure included depressive episodes with psychotic symptoms (*ICD-10* codes F32.3 and F33.3) and psychosis has been associated with neighborhood context,^54,55^ we conducted a post hoc sensitivity analysis excluding these individuals and their matched controls to evaluate their influence on the associations (see eMethods in Supplement 1). Statistical significance was defined as 2-sided *P* < .05.

## Results

In the general population–matched sample of 571 470 individuals, 95 245 (36 297 male individuals [38.1%]) had an outpatient depression diagnosis between 2001 and 2020 (Table 2). The median (IQR) age at first diagnosis was 20 (17-24) years. In the sibling-matched sample of 85 170 individuals, 42 585 (16 058 male individuals [37.7%]) had an outpatient depression diagnosis between 2001 and 2020. The median (IQR) age at first diagnosis was 19 (17-23) years. In both matched samples, individuals with depression had higher rates of violent and nonviolent convictions across all neighborhood types (see Table 2 for baseline descriptive data).

**Table 2.** Descriptive Statistics for the General Population–Matched and Sibling-Matched Samples

Characteristic	Participants, No. (%)														
	Total sample			Resource-limited neighborhoods			Rural low-diversity neighborhoods			Urban professional neighborhoods			Urban affluent neighborhoods		
	Total	Cases	Controls	Total	Cases	Controls	Total	Cases	Controls	Total	Cases	Controls	Total	Cases	Controls
General population–matched sample															
No. of participants	571 470	95 245	476 225	52 548	8758	43 790	262 662	43 777	218 885	131 310	21 885	109 425	124 950	20 825	104 125
Age at first diagnosis, median (IQR), y	NA	20 (17-24)	NA	NA	21 (18-25)	NA	NA	19 (17-24)	NA	NA	22 (19-26)	NA	NA	18 (17-22)	NA
Sex															
Male	217 782 (38.1)	36 297 (38.1)	181 485 (38.1)	20 274 (38.6)	3379 (38.6)	16 895 (38.6)	101 646 (38.7)	16 941 (38.7)	84 705 (38.7)	48 522 (37.0)	8087 (37.0)	40 435 (37.0)	47 340 (37.9)	7890 (37.9)	39 450 (37.9)
Female	353 688 (61.9)	58 948 (61.9)	294 740 (61.9)	32 274 (61.4)	5379 (61.4)	26 895 (61.4)	161 016 (61.3)	26 836 (61.3)	134 180 (61.3)	82 788 (63.0)	13 798 (63.0)	68 990 (63.0)	77 610 (62.1)	12 935 (62.1)	64 675 (62.1)
Criminal convictions															
Violent	8499 (1.5)	3079 (3.2)	5420 (1.1)	1469 (2.8)	415 (4.7)	1054 (2.4)	4423 (1.7)	1691 (3.9)	2732 (1.2)	1385 (1.1)	516 (2.4)	869 (0.8)	1222 (1.0)	457 (2.2)	765 (0.7)
Nonviolent	32 246 (5.6)	9912 (10.4)	22 334 (4.7)	5011 (9.5)	1206 (13.8)	3805 (8.7)	15 906 (6.1)	5216 (11.9)	10 690 (4.9)	5616 (4.3)	1800 (8.2)	3816 (3.5)	5713 (4.6)	1690 (8.1)	4023 (3.9)
Psychiatric disorders															
Substance use disorders	36 721 (6.4)	16 196 (17.0)	20 525 (4.3)	4671 (8.9)	1907 (21.8)	2764 (6.3)	15 710 (6.0)	7131 (16.3)	8579 (3.9)	9378 (7.1)	4123 (18.8)	5255 (4.8)	6962 (5.6)	3035 (14.6)	3927 (3.8)
Attention-deficit/hyperactivity disorder	47 347 (8.3)	25 880 (27.2)	21 467 (4.5)	4895 (9.3)	2513 (28.7)	2382 (5.4)	23 314 (8.9)	12 648 (28.9)	10 666 (4.9)	8.948 (6.8)	5101 (23.3)	3847 (3.5)	10 190 (8.2)	5618 (27.0)	4572 (4.4)
Prior convictions	38 543 (6.7)	10 503 (11.0)	28 040 (5.9)	6367 (12.1)	1484 (16.9)	4883 (11.2)	17 043 (6.5)	5026 (11.5)	12 017 (5.5)	9349 (7.1)	2448 (11.2)	6901 (6.3)	5784 (4.6)	1545 (7.4)	4239 (4.1)
Sibling-matched sample															
No. of participants	85 170	42 585	42 585	5186	2593	2593	43 156	21 578	21 578	15 594	7797	7797	21 234	10 617	10 617
Age at first diagnosis, median (IQR), y	NA	19 (17-23)	NA	NA	19 (17-23)	NA	NA	19 (17-23)	NA	NA	21 (18-25)	NA	NA	18 (16-20)	NA
Sex															
Male	39 397 (46.3)	16 058 (37.7)	23 339 (54.8)	2415 (37.9)	983 (37.9)	1432 (55.2)	20 093 (37.5)	8091 (37.5)	12 002 (55.6)	7079 (38.2)	2976 (38.2)	4103 (52.6)	9810 (37.8)	4008 (37.8)	5802 (54.6)
Female	45 773 (53.7)	26 527 (62.3)	19 246 (45.2)	2771 (62.1)	1610 (62.1)	1161 (44.8)	23 063 (62.5)	13 487 (62.5)	9576 (44.4)	8515 (61.8)	4821 (61.8)	3694 (47.4)	11 424 (62.8)	6609 (62.8)	4815 (45.4)
Criminal convictions															
Violent	2086 (2.4)	1231 (2.9)	855 (2.0)	258 (5.0)	141 (5.4)	117 (4.5)	1249 (2.9)	734 (3.4)	515 (2.4)	252 (1.6)	150 (1.9)	102 (1.3)	327 (1.5)	206 (1.9)	121 (1.1)
Nonviolent	7130 (8.4)	4119 (9.7)	3011 (7.1)	703 (13.6)	395 (15.2)	308 (11.9)	4016 (9.3)	2333 (10.8)	1683 (7.8)	1031 (6.6)	599 (7.7)	432 (5.5)	1380 (6.5)	792 (7.5)	588 (5.5)

Abbreviation: NA, not applicable.

Conditional logistic regression analyses on the general population–matched sample showed significantly increased odds of both violent and nonviolent convictions among individuals with depression across all neighborhood types (Table 3 and Table 4). Wald tests on unadjusted and fully adjusted models indicated that only resource-limited neighborhoods differed significantly from the rural low-diversity neighborhood for violent criminal convictions (OR, 3.27; 95% CI, 3.07-3.48), with a lower OR observed in resource-limited neighborhoods (OR, 2.08; 95% CI, 1.85-2.34). For nonviolent criminal convictions, both resource-limited (OR, 1.75; 95% CI, 1.63-1.89) and urban affluent (OR, 2.28; 95% CI, 2.15-2.43) neighborhoods showed significantly lower ORs compared with rural low-diversity neighborhoods (OR, 2.76; 95% CI, 2.67-2.86). After full adjustment, associations were attenuated but remained significant in all neighborhood types except resource-limited neighborhoods (violent conviction OR, 1.14 [95% CI, 0.97-1.33]; nonviolent conviction OR, 1.01 [95% CI, 0.92-1.11]).

**Table 3.** Associations Between Depression and Violent Criminal Convictions

Neighborhood type	General population–matched sample							Sibling-matched sample		
	No. of participants	OR (95% CI)				*P* value		No. of participants	Unadjusted OR (95% CI)	*P* value^f^
		Unadjusted	Adjusted^a^	Adjusted^b^	Adjusted^c^	Unadjusted^d^	Adjusted^e^			
Resource limited	52 548	2.08 (1.85-2.34)	1.79 (1.59-2.05)	1.26 (1.08-1.46)	1.14 (0.97-1.33)	<.001	.004	5186	1.26 (0.96-1.65)	.53
Rural low-diversity	262 662	3.27 (3.07-3.48)	2.74 (2.55-2.94)	2.00 (1.85-2.16)	1.51 (1.39-1.65)	[Reference]	[Reference]	43 156	1.50 (1.33-1.69)	.93
Urban professional	131 310	3.08 (2.76-3.44)	2.62 (2.31-2.97)	1.81 (1.57-2.09)	1.49 (1.28-1.73)	>.99	.86	15 594	1.52 (1.17-1.98)	.89
Urban affluent	124 950	3.11 (2.76-3.50)	2.76 (2.43-3.15)	1.96 (1.70-2.27)	1.52 (1.30-1.78)	>.99	.95	21 234	1.81 (1.43-2.30)	.23

Abbreviation: OR, odds ratio.

^a^ Adjusted for prior convictions.

^b^ Also adjusted for substance use disorder.

^c^ Also adjusted for attention-deficit/hyperactivity disorder.

^d^ Wald test was used to compare unadjusted estimates for each neighborhood type with the rural low-diversity reference, with Bonferroni-adjusted *P* values.

^e^ Wald test was used to compare fully adjusted estimates for each neighborhood type with the rural low-diversity reference, with Bonferroni-adjusted *P* values.

^f^ Wald test was used to compare fully adjusted estimates from the general population-matched sample with unadjusted estimates from the sibling-matched sample, conducted separately within each neighborhood type.

**Table 4.** Associations Between Depression and Nonviolent Criminal Convictions

Neighborhood type	General population–matched sample							Sibling-matched sample		
	No. of participants	OR (95% CI)				*P* value		No. of participants	Unadjusted OR (95% CI)	*P* value^f^
		Unadjusted	Adjusted^a^	Adjusted^b^	Adjusted^c^	Unadjusted^d^	Adjusted^e^			
Resource limited	52 548	1.75 (1.63-1.89)	1.53 (1.41-1.66)	1.11 (1.01-1.21)	1.01 (0.92-1.11)	<.001	<.001	5186	1.38 (1.17-1.64)	.002
Rural low-diversity	262 662	2.76 (2.67-2.86)	2.38 (2.29-2.48)	1.78 (1.71-1.86)	1.49 (1.42-1.56)	[Reference]	[Reference]	43 156	1.49 (1.39-1.60)	>.99
Urban professional	131 310	2.59 (2.44-2.75)	2.25 (2.11-2.41)	1.56 (1.45-1.68)	1.35 (1.25-1.46)	.23	.10	15 594	1.48 (1.30-1.69)	.24
Urban affluent	124 950	2.28 (2.15-2.43)	2.06 (1.93-2.19)	1.50 (1.39-1.61)	1.28 (1.18-1.38)	<.001	.002	21 234	1.42 (1.26-1.59)	.13

Abbreviation: OR, odds ratio.

^a^ Adjusted for prior convictions.

^b^ Also adjusted for substance use disorder.

^c^ Also adjusted for attention-deficit/hyperactivity disorder.

^d^ Wald test was used to compare unadjusted estimates for each neighborhood type with the rural low-diversity reference, with Bonferroni-adjusted *P* values.

^e^ Wald test was used to compare fully adjusted estimates for each neighborhood type with the rural low-diversity reference, with Bonferroni-adjusted *P* values.

^f^ Wald test was used to compare fully adjusted estimates from the general population-matched sample with unadjusted estimates from the sibling-matched sample, conducted separately within each neighborhood type.

### Unmeasured Familial Confounding

Estimates from the sibling-matched sample remained largely consistent with fully adjusted general population–matched estimates for both violent and nonviolent convictions (eg, violent convictions in rural low-diversity neighborhoods: sibling-matched OR, 1.50 [95% CI, 1.33-1.69] vs general population-matched OR, 1.51 [95% CI, 1.39-1.65]). The Figure presents forest plots illustrating these estimates, alongside unadjusted general population–matched estimates for comparability. Wald test comparisons confirmed these findings (Table 3 and Table 4). The only significant difference was observed in resource-limited neighborhoods for nonviolent criminal convictions, where the unadjusted OR (1.38; 95% CI, 1.17-1.64) significantly exceeded the fully adjusted OR (1.01; 95% CI, 0.92-1.11).

**Figure.**
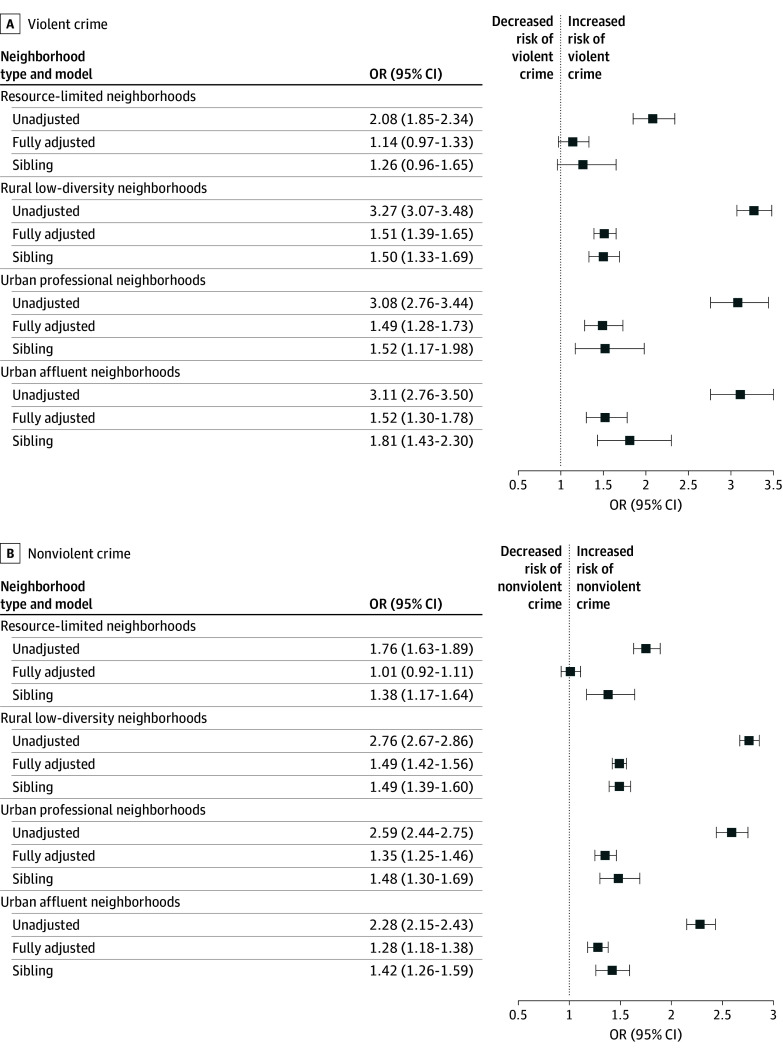
Associations Between Depression and Criminal Convictions Across Neighborhood Classes Forest plots show odds ratios (ORs) for violent (A) and nonviolent (B) criminal convictions. Unadjusted and fully adjusted estimates are presented for the general population–matched sample. Fully adjusted models control for prior convictions, substance use disorder, and attention-deficit/hyperactivity disorder.

### Sensitivity Analysis

Among individuals without comorbid SUD, associations between depression and criminal convictions were attenuated but generally persisted across all neighborhood types, except in resource-limited neighborhoods (eTables 1-3 in Supplement 1). In a separate post hoc sensitivity analysis excluding individuals with depressive episodes with psychotic symptoms (*ICD-10* codes F32.3 and F33.3) and their matched controls, results closely mirrored the main findings (eTables 4 and 5 in Supplement 1).

## Discussion

In this nationwide cohort study, we examined whether the association between depression and violent and nonviolent criminal convictions varied across distinct neighborhood types by comparing 95 245 cases with a depression diagnosis with 476 225 controls without depression matched on age, sex, and neighborhood type and following individuals over time. Using sibling analyses, we also assessed the extent to which unmeasured familial factors contributed to these associations. To our knowledge, this is the first study to examine how these associations differed by distinct neighborhood types. This study advances previous research in several important ways.

First, individuals with depression had significantly higher odds of violent and nonviolent convictions across all neighborhood types (resource-limited, rural low-diversity, urban professional, and urban affluent) in unadjusted models. This pattern remained largely consistent, although attenuated, in fully adjusted models. Wald tests confirmed that associations varied across neighborhood types, with lower odds in resource-limited neighborhoods compared with rural low-diversity neighborhoods for both crime types, and in urban affluent neighborhoods for nonviolent criminal convictions. Across all models, individuals in resource-limited neighborhoods consistently had lower odds than those in other neighborhood types. This overall trend was unexpected and contrasts with General Strain Theory,^12^ given that this neighborhood type was characterized by socioeconomic deprivation and ethnic heterogeneity, factors associated with elevated depression rates and increased crime.^17,18,21,22,25,27^ Although resource-limited neighborhoods had the highest conviction frequencies, the smaller association with depression suggests that depression was a less important risk factor for crime in these neighborhoods. Alternatively, this pattern could reflect potential underreporting and selection bias, including barriers to accessing mental health services,^56^ affecting detection and treatment of depression, leading to a potential underestimation of the association. These findings should also be interpreted in light of the inclusion of SUD as a covariate. Although SUD is associated with both depression and crime,^49,50^ it may lie on the causal pathway between them, complicating interpretation due to possible overadjustment. However, sensitivity analyses excluding individuals with comorbid SUD indicated that elevated odds remained, suggesting a potentially independent association.

Second, unmeasured familial confounding only partially explained the association between depression and criminal convictions across neighborhood types. Sibling-matched estimates largely resembled fully adjusted general population–matched estimates. The similarity may reflect overlap between adjusted covariates, including SUD and ADHD, and familial factors inherently accounted for in sibling comparisons. This aligns with evidence that both SUD and ADHD are highly heritable.^57,58,59^ To ensure comparability with prior research, we presented unadjusted, fully adjusted, and sibling-matched estimates, conducting statistical comparisons between the latter two. However, these estimates should not be interpreted as directly comparable because of differences in sample composition and statistical precision. Although some research suggests that heritable influences on depression increased with neighborhood deprivation,^32^ our results support evidence that heritable contributions to criminal behavior are greater in higher socioeconomic neighborhoods,^31^ consistent with the lesser familial confounding observed in resource-limited neighborhoods. This is in line with the social push hypothesis, which posits that the effect of biological factors is attenuated in adverse environments where social risk factors are more critical.^60,61^

Overall, our findings indicate that the association between depression and criminal convictions is heterogeneous across neighborhood types, underscoring the relevance of considering neighborhood context in future research. These patterns may also inform crime-prevention efforts. We observed that the depression-crime association was smaller and less confounded by familial factors in resource-limited neighborhoods, suggesting that contextual risk may play a comparatively larger role in these settings. Place-based approaches that strengthen social and structural resources in disadvantaged areas may, therefore, be particularly relevant, consistent with evidence that simple, scalable interventions can reduce externalizing behavior in young individuals.^62^ Although our comprehensive measure of neighborhood social structure may reduce the ability to disentangle the contribution of specific neighborhood factors,^36^ it enhances ecological validity and may support the practical applicability of our findings for designing place-based interventions. Following from our work, we emphasize the need for further research into mechanisms, sex and age differences, comorbidity of other psychiatric disorders, and the temporal sequence of these associations. Our observational design cannot estimate causal risk ratios or average causal effects. Future work using causal inference methods is, therefore, warranted. Future studies should also apply advanced methods, such as familial aggregation analyses or genetically sensitive designs, to further investigate familial influences.

### Limitations

Several limitations warrant consideration. First, Swedish national registers lack primary care data, likely underestimating depression prevalence, as milder cases are often treated outside specialist care.^63^ Second, we examined individual-level convictions but lacked data on neighborhood crime rates or crime locations. Although we indexed residential neighborhoods, individuals are exposed to multiple environments. Third, generalizability may be limited, as data and neighborhood types are specific to Sweden. Fourth, low frequencies required combining 2 smaller neighborhood types into the resource-limited type. Although these differed on some indicators (eg, urbanicity), both represented socioeconomically deprived neighborhood types. Their low frequencies may reflect Sweden’s comprehensive welfare system, which helps mitigate social inequalities. Furthermore, the neighborhood type labeled rural low-diversity was characterized by low ethnic heterogeneity and predominantly rural municipalities, as defined by Statistics Sweden. Although the label reflects the dominant features of the type, it may not capture its full nuance. These label choices should be interpreted within the context of the classification method. Additional details are provided in eMethods in Supplement 1.

## Conclusions

In a cohort study of the Swedish general population, we used a comprehensive measure of neighborhood social structure and a matched design and found that the association between depression and criminal convictions varied across neighborhood types. This association was lowest in resource-limited neighborhoods, and familial confounding only partially explained the association. Our results highlight the relevance of neighborhood context in the link between depression and crime and underscore the need for further research into mechanisms underlying this association.

## Notes

Supplement 1. eMethods. eFigure 1. Flowchart illustrating the matching procedure for the general population-matched sample eFigure 2. Flowchart illustrating the matching procedure for the sibling-matched sample eTable 1. Descriptive statistics for the general population-matched sample with comorbid SUD eTable 2. Descriptive statistics for the general population-matched sample without comorbid SUD eTable 3. Association between depression and violent and non-violent criminal convictions, stratified by neighborhood type and comorbid SUD eTable 4. Descriptive statistics for the general population-matched sample, excluding cases with depressive episodes with psychotic symptoms and their matched controls eTable 5. Associations between depression and violent and non-violent criminal convictions across neighborhood types, excluding cases with depressive episodes with psychotic symptoms and their matched controls eReferences

Supplement 2. Data Sharing Statement
