# Supplementary material for: Depression and Crime Across Different Neighborhoods in the Swedish General Population
Source: JAMA Netw Open. 2026 Feb 3;9(2):e2557546. doi: 10.1001/jamanetworkopen.2025.57546 (PMC12869338; doi:10.1001/jamanetworkopen.2025.57546)
Supplement: Supplement 1. — eMethods. eFigure 1. Flowchart illustrating the matching procedure for the general population-matched sample eFigure 2. Flowchart illustrating the matching procedure for the sibling-matched sample eTable 1. Descriptive statistics for the general population-matched sample with comorbid SUD eTable 2. Descriptive statistics for the general population-matched sample without comorbid SUD eTable 3. Association between depression and violent and non-violent criminal convictions, stratified by neighborhood type and comorbid SUD eTable 4. Descriptive statistics for the general population-matched sample, excluding cases with depressive episodes with psychotic symptoms and their matched controls eTable 5. Associations between depression and violent and non-violent criminal convictions across neighborhood types, excluding cases with depressive episodes with psychotic symptoms and their matched controls eReferences [file jamanetwopen-e2557546-s001.pdf]

Supplemental Online Content

Tayebi N, Andersson A, Fazel S, Larsson H, Evans B, Tuvblad C. Depression and crime across different neighborhoods in the Swedish general population. *JAMA Netw Open*. 2026;9(2):e2557546. doi:10.1001/jamanetworkopen.2025.57546

eMethods

eFigure 1. Flowchart illustrating the matching procedure for the general population-matched sample

eFigure 2. Flowchart illustrating the matching procedure for the sibling-matched sample

eTable 1. Descriptive statistics for the general population-matched sample with comorbid SUD

eTable 2. Descriptive statistics for the general population-matched sample without comorbid SUD

eTable 3. Association between depression and violent and non-violent criminal convictions, stratified by neighborhood type and comorbid SUD

eTable 4. Descriptive statistics for the general population-matched sample, excluding cases with depressive episodes with psychotic symptoms and their matched controls

eTable 5. Associations between depression and violent and non-violent criminal convictions across neighborhood types, excluding cases with depressive episodes with psychotic symptoms and their matched controls

eReferences

This supplemental material has been provided by the authors to give readers additional information about their work.

© 2026 Tayebi N et al. *JAMA Network Open*.

eMethods.

Data Sources. We linked multiple Swedish population-based registries using the unique personal identity number assigned to all residents at birth or immigration.[1] This linkage was approved by the Swedish Ethical Review Authority (2022-06204-02). The following registries were used to identify the study cohort: (a) Total Population Register (TPR), with demographic data for all Swedish residents born in Sweden since 1932.[2] (b) National Patient Register (NPR), including inpatient (since 1973) and outpatient (since 2001) diagnoses recorded according to the Swedish version of the International Classification of Diseases (ICD).[3] (c) National Crime Register (NCR), which includes lower court conviction data since 1973 for individuals aged 15 or older, the minimum age of criminal responsibility in Sweden. (d) Multi-Generation Register (MGR), which includes information on family relations, thus enabling identification of relatives.[4] (e) Longitudinal Integration Database for Health Insurance and Labor Market Studies (LISA), with annual employment, education, and income data since 1990.[5] (f) Demographic Statistical Areas (DeSO), providing annual data on neighborhood residence and area characteristics.[6]

Study Population. Using the TPR, we identified all individuals born between 1986 and 2005 (*N* = 2,875,441) to ensure they were at least 15 years old when outpatient data became available. Exclusions were made for individuals who had ever immigrated or emigrated (*n* = 941,960) or died (*n* = 13,067) before the age of 15, as the follow-up period commenced only after age 15. To mitigate the risk of reverse causation, individuals with recorded inpatient episodes of depression were excluded (*n* = 35,326). Individuals who received an outpatient diagnosis of depression prior to attaining the age of 15 were also excluded from the analyses (*n* = 9,503). We additionally excluded individuals with diagnoses of bipolar disorder (*n* = 15,300), schizophrenia, or schizophrenia spectrum disorders (*n* = 8,238) in either in- or outpatient care, due to the association between these psychiatric disorders and increased risk of criminal behavior.[7,8] Lastly, individuals with missing information about neighborhood type across all timepoints were excluded (*n* = 1,905). This yielded a total cohort of 1,850,142 individuals, of which 52% were males and 48% females. Since outpatient care data in the NPR became available in 2001,[3,9] our follow-up began on January 1, 2001. Specifically, the follow-up initiation was set at the commencement of 2001 or later, aligning with the attainment of age 15, the minimum age of criminal responsibility in Sweden. Participants were subsequently followed until the end of the follow-up period (December 31, 2020) or death, whichever occurred first. In total, 7,277 individuals (0.4% of the final cohort) died during the follow-up period.

© 2026 Tayebi N et al. *JAMA Network Open*.

Missing Data. This study used data from several Swedish population-based registers with high coverage and validity. Depression diagnoses, used as the exposure, were identified through the NPR, as were the covariates substance use disorder (SUD) and attention-deficit/hyperactivity disorder (ADHD). A recent validation study has reported generally high diagnostic accuracy for psychiatric disorders in the NPR, with particularly strong validity for most diagnostic codes.[9] Some missingness has been observed, primarily among private healthcare providers and for specific variables such as drug administration. According to an earlier validation study, the proportion of missing primary diagnoses in inpatient psychiatric care was approximately 3.1%.[3]

The outcomes, violent and non-violent criminal convictions, were identified using the NCR. The covariate, prior convictions, was also derived from this register. This NCR includes all individuals convicted in a Swedish court from age 15 onward but does not capture unreported or unprosecuted offenses. While both the date of crime and date of conviction are recorded, the conviction date was used in this study due to greater completeness. In our full cohort, 0.4% of individuals with a conviction date were missing a corresponding date of crime. Neighborhood social structure was measured using pre-existing neighborhood classifications.[10] Individuals missing neighborhood type for all follow-up years (n = 1,905) were excluded prior to final cohort construction.

Lastly, parental linkage for the sibling analysis was based on the MGR, which includes information on mothers and fathers for approximately 97% and 95% of index persons, respectively.[4] In our final cohort, 11,712 individuals had missing parental information. These individuals were retained in the general population-matched sample, where parental linkage was not required, but were excluded from the sibling-matched sample, where identification of siblings depended on parental information.

Neighborhood Social Structure. In previous research, neighborhood social structure was characterized using latent profile analyses (LPA).[10] The analysis included 6,236 distinct neighborhoods classified according to the DeSO system and utilized indicators from multiple national registers (DeSO, TPR, LISA, and MGR) to capture four key dimensions: socioeconomic conditions, ethnic heterogeneity, residential instability, and urbanicity. The indicators were aggregated at the neighborhood level from 1991 to 2020. Urbanicity was assessed using Statistics Sweden's classification of municipalities based on population density and proximity to urban centers, and this information was used to inform the labeling of neighborhoods. For example, neighborhoods classified as "rural" had low population density and were not part of metropolitan or urban-adjacent areas. Of the 6,236 DeSO neighborhoods initially available, two were excluded due to complete missingness on all indicators for the timepoints relevant to the present study, resulting in a final analytic sample of 6,234 distinct neighborhoods. LPA results identified five neighborhood types: (a) *Rural resource-limited neighborhoods* (3% of neighborhoods) were characterized by the lowest education and income levels, the highest scores on residential instability, proportion of social benefit recipients, and proportion of unmarried individuals, and second-highest scores on ethnic heterogeneity. (b) *Urban high-diversity neighborhoods* (9% of neighborhoods) were characterized by the highest score on ethnic heterogeneity and the second-lowest scores on income, education, and proportion of social benefit recipients. (c) *Rural low-diversity neighborhoods* (46% of neighborhoods) were characterized by the lowest scores on ethnic

© 2026 Tayebi N et al. *JAMA Network Open*.

heterogeneity, urbanicity, and residential instability. (d) *Urban professional neighborhoods* (19% of neighborhoods) were characterized by the highest score on urbanicity, and the second-highest scores on residential instability, proportion of unmarried individuals, and education. Lastly, (e) *Urban affluent neighborhoods* (23% of neighborhoods) were characterized by the highest income and education levels, and the lowest scores on residential instability, proportion of unmarried individuals, and proportion of social benefits recipients. The neighborhood classification demonstrated good model fit and conceptual validity. Comprehensive model evaluation, including details on all fit indices, confirmatory analyses, variable specification, classification procedures, and the resulting types, is available in the published methodological work.[10]

The measures were aggregated the to six time points by combining data into five-year intervals (1991-1995, 1996-2000, 2001-2005, 2006-2010, 2011-2015, and 2016-2020).[10] Latent transition probabilities for neighborhood types across timepoints indicated high stability, ranging from 88% to 100%. As outpatient data in the NPR became available in 2001,[3,9] only the time-points after 2001 were included in the present study. Moreover, due to limited samples, the two smallest and most similar neighborhoods, *Rural resource-limited* and *Urban high-diversity* neighborhoods, were combined into a single neighborhood type in the present study, referred to as *Resource-limited neighborhoods*. This type represents neighborhoods with the highest socioeconomic disadvantage, mid-to-high ethnic heterogeneity, and intermediate urbanicity, neither clearly urban nor rural. We acknowledge that the name "resource-limited" better reflects this group's defining feature (socioeconomic disadvantage). Similarly, although the "rural low-diversity" type was labeled based on its low urbanicity and ethnic homogeneity, we recognize that it includes a large portion of the Swedish population, as it reflects the predominance of smaller towns and rural municipalities.

Matching procedure. Before conducting the analyses, we constructed two distinct sets of matched groups. In the first set, individuals diagnosed with depression (cases) were matched to undiagnosed controls from the general population. In the second set, individuals with depression (cases) were matched to undiagnosed siblings (controls). The matched groups were created using SAS software version 9.4.

Neighborhood type was measured in predefined five-year intervals (2001–2005, 2006–2010, 2011–2015, 2016–2020),[10] with each individual assigned the neighborhood type in which they resided most often during that interval. To align with the structure of the neighborhood types, which were categorized into four five-year intervals, we also created five-year intervals for birth year and diagnosis date to facilitate the matching process. Birth years were grouped into 1986–1990, 1991–1995, 1996–2000, and 2001–2005, referred to as *birth year periods*. Similarly, diagnosis dates (the date of the first outpatient diagnosis) were grouped into 2001–2005, 2006–2010, 2011–2015, and 2016–2020, referred to as *depression date periods*.

General Population-Matched Sample. We matched each individual with an outpatient diagnosis of depression (cases) with a unique subset of undiagnosed controls from the general population individually by birth year period, sex, and neighborhood type. For the cases, the neighborhood type corresponding to the depression date period was exclusively used in the matching process. For controls, who lacked a depression date and could have multiple neighborhood types between 2001 and 2020, a random selection process was utilized to retain

© 2026 Tayebi N et al. *JAMA Network Open*.

a single five-year interval per control prior to matching. This was necessary to ensure that each control contributed only one five-year interval and one neighborhood type to the matching process. Without this restriction, a single individual could potentially serve as a control for multiple cases by contributing with different neighborhood types from different intervals. Random selection was used to avoid introducing systematic bias. The neighborhood type for the selected interval was already pre-assigned based on the individual's most common residence during that five-year period, and this type served as the basis for matching to cases. Controls were matched to cases when their retained neighborhood type corresponded to the case's neighborhood type during the depression date period. To ensure an equal distribution of controls for each case, and to maximize the available number of controls, the present study relied on five unique age-, sex-, and neighborhood type-matched controls per case. To determine the subset of five controls selected for each case, a random selection process using the RANUNI function in SAS was implemented within the pool of relevant controls. Following the matching of cases and undiagnosed population controls, the sample consisted of a total of 571,470 individuals, where 95,245 were cases with a depression diagnosis and 476,225 were undiagnosed population controls. See eFigure 1 for a flowchart detailing the matching procedure for the general population-matched sample.

Sibling-Matched Sample. To create a sibling-matched sample, we matched individuals with an outpatient diagnosis of depression (cases) to their undiagnosed full siblings (controls). Sibling relationships were identified using parental data retrieved from the MGR. Several exclusions were applied to the cohort prior to matching. We excluded individuals with missing parental data (n = 11,718), individuals without siblings (n = 546,982), individuals in sibling groups without any cases of depression (n = 1,154,711), and individuals in sibling groups where all siblings had a depression diagnosis (n = 4,807). Following these exclusions, 131,924 individuals remained eligible for the sibling match.

The match was conducted on a 1:1 ratio of depressed to undiagnosed siblings within sibling groups and was further restricted by neighborhood type. Specifically, only undiagnosed siblings (controls) who shared the same neighborhood type as their depressed sibling (case) during the depression date period were eligible for matching. In sibling groups with unequal numbers of depressed and undiagnosed siblings, random selection using the RANUNI function in SAS was applied to maintain the 1:1 matching ratio. Following the matching of cases with depression and their undiagnosed full siblings, the sample consisted of a total of 85,170 individuals, where 42,585 were cases with a depression diagnosis and 42,585 were undiagnosed full sibling controls. See eFigure 2 for a flowchart detailing the matching procedure for the sibling-matched sample.

For both sets of matched groups, we obtained information on the date of the first outpatient depression diagnosis for each case from the NPR. In the general population-matched groups, this diagnosis date was uniformly assigned to the unique subset of undiagnosed age-, sex-, and neighborhood type controls matched to each case. Similarly, in the sibling-matched groups, the diagnosis date was assigned to the undiagnosed full sibling matched to each case. This ensured a uniform starting point for follow-up between cases and controls in both matched groups. Convictions predating the diagnosis date were considered and treated as a covariate referred to as *prior convictions*. Convictions occurring after the diagnosis date were treated as either the outcome violent criminal convictions or non-violent criminal convictions.

© 2026 Tayebi N et al. *JAMA Network Open*.

Sensitivity Analysis. Substance-related problems are associated with both depression and crime and has also been linked to neighborhood social structure.[11–14] To clarify its role in the association between depression and crime across neighborhood types, we conducted a sensitivity analysis by further stratifying the general population-matched sample based on SUD status, examining whether associations persisted irrespective of comorbidity. Using the general population-matched sample, we identified all individuals with comorbid SUD through inpatient or outpatient diagnoses in the NPR using the following ICD codes: ICD-8: 303, 304; ICD-9: 303, 304, 305.1, 305.9; ICD-10: F10-F19. Individuals were considered to have comorbid SUD if they had received at least one such diagnosis at any time, regardless of whether it occurred before or after the depression diagnosis. Individuals without any such diagnosis during follow-up were considered not to have comorbid SUD. A total of 16,196 individuals with depression and comorbid SUD were identified, together with their five unique age-, sex-, and neighborhood type-matched undiagnosed controls, they comprised a sample of 97,176 (of which 80,980 were controls). Additionally, 79,049 individuals with depression but without comorbid SUD were identified, along with their five unique matched controls, they formed a sample of 474,294 (of which 395,245 were controls). For descriptive statistics on both samples, see eTable 1 and eTable 2. Results are shown in eTable 3.

Neighborhood deprivation and ethnic density have been associated with psychosis,[15,16] and the ICD-10 codes used to define depression in this study (F32.3 and F33.3) include severe depressive episodes with psychotic symptoms. To assess whether these cases influenced the observed associations between depression and criminal convictions across neighborhood types, we conducted a post hoc sensitivity analysis excluding individuals with depressive episodes with psychotic symptoms and their matched controls from the general population-matched sample. A total of 348 individuals with ICD-10 F32.3/F33.3 were identified, along with their 1,740 unique age-, sex-, and neighborhood type-matched controls. The resulting analytic sample therefore excluded these 2,088 individuals. Descriptive statistics for the sample are presented in eTable 4, and the associations between depression and violent and non-violent criminal convictions across neighborhood types are shown in eTable 5.

© 2026 Tayebi N et al. *JAMA Network Open*.

eFigure 1. Flowchart illustrating the matching procedure for the general population-matched sample.

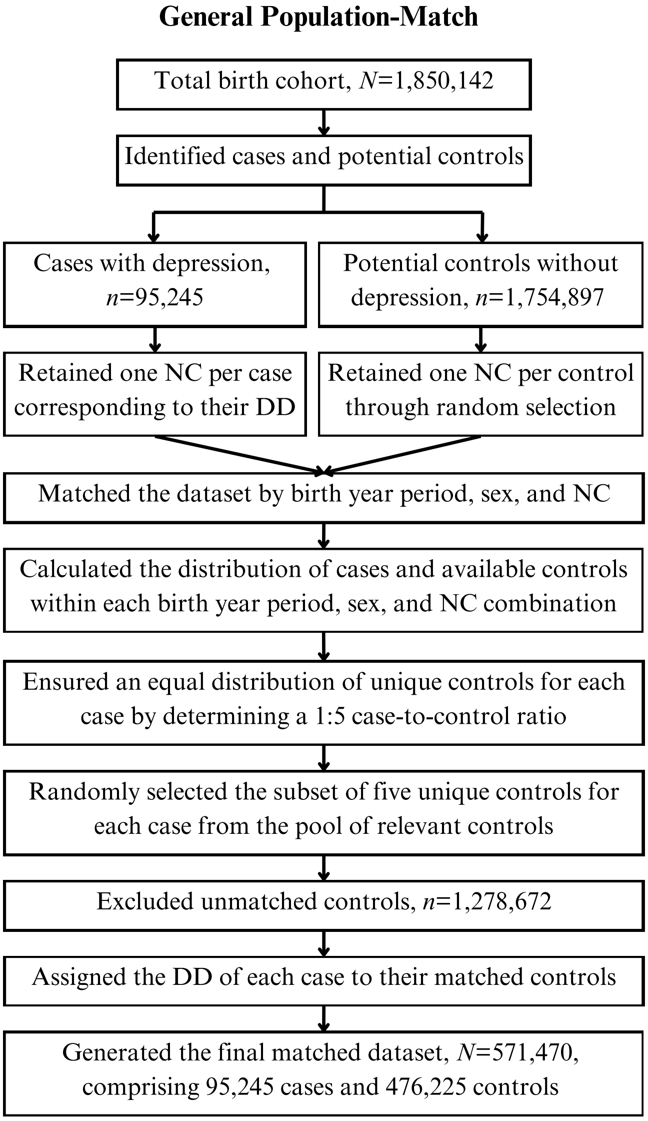

DD = date of first outpatient depression diagnosis. NC = neighborhood type.

© 2026 Tayebi N et al. *JAMA Network Open*.

eFigure 2. Flowchart illustrating the matching procedure for the sibling-matched sample.

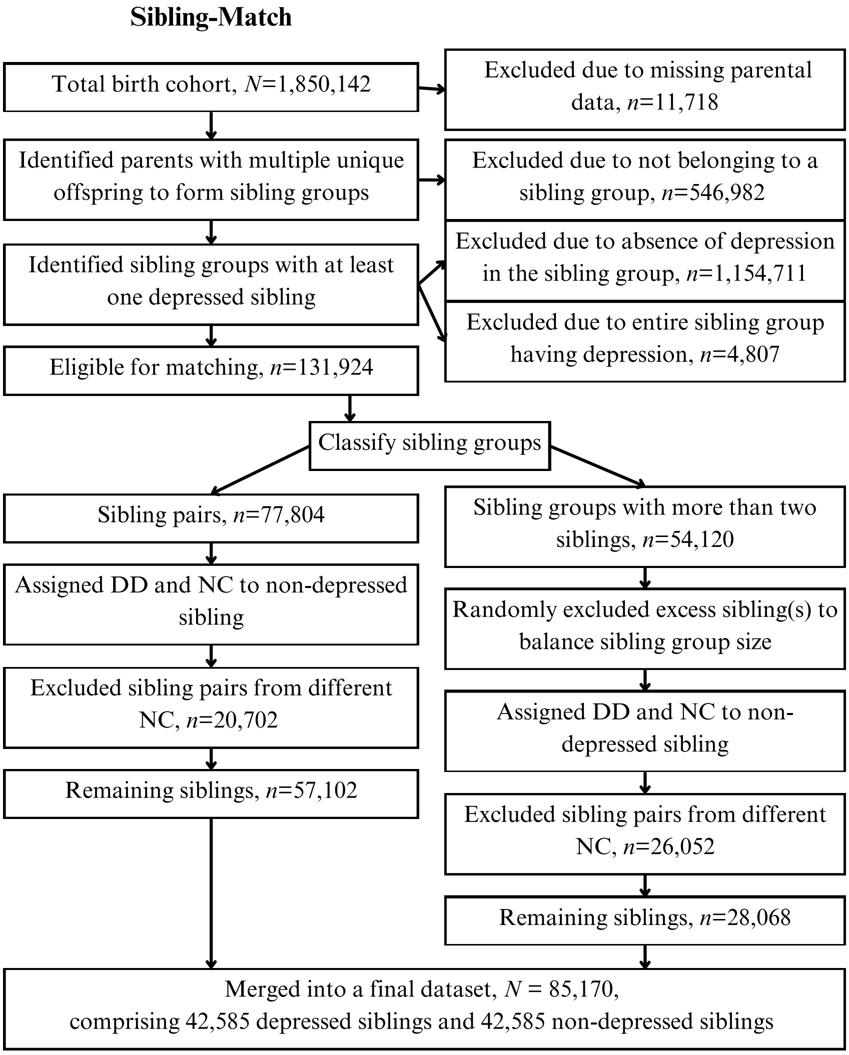

DD = date of first outpatient depression diagnosis. NC = neighborhood type.

© 2026 Tayebi N et al. *JAMA Network Open*.

eTable 1. Descriptive statistics for the general population-matched sample with comorbid SUD.

	No. (%)										
	**Total sample** *N*=**97,176**			**Resource-limited neighborhoods** *N*=**11,442**		**Rural low-diversity neighborhoods** *N*=**42,786**		**Urban professional neighborhoods** *N*=**24,738**		**Urban affluent neighborhoods** *N*=**18,210**	
	Total	Cases *n*=16,196	Controls *n*=80,980	Cases, *n*=1,907	Controls *n*=9,535	Cases *n*=7,131	Controls *n*=35,655	Cases *n*=4,123	Controls *n*=20,615	Cases *n*=3,035	Controls *n*=15,175
**Age at first diagnosis, y, median (IQR)**	NA	21 (18-25)	NA	22 (19-26)	NA	21 (18-24)	NA	23 (19-26)	NA	19 (17-23)	NA
**Sex**											
Male	45,966 (47.3%)	7,661 (47.3%)	38,305 (47.3%)	901 (47.2%)	4,505 (47.2%)	3,454 (48.4%)	17,270 (48.4%)	1,878 (45.5%)	9,390 (45.5%)	1,428 (47.1%)	7,140 (47.1%)
Female	51,210 (52.7%)	8,535 (52.7%)	42,675 (52.7%)	1,006 (52.8%)	5,030 (52.8%)	3,677 (51.6%)	18,385 (51.6%)	2,245 (54.5%)	11,225 (54.5%)	1,607 (52.9%)	8,035 (52.9%)
**Criminal convictions**											
Violent	2,901 (3.0%)	1,745 (10.8%)	1,156 (1.4%)	256 (13.4 %)	290 (3.0%)	909 (12.7%)	545 (1.5%)	330 (8.0%)	180 (0.9%)	250 (8.2%)	141 (0.9%)
Non-violent	9,626 (9.9%)	5,174 (31.9%)	4,452 (5.5%)	705 (37.0%)	960 (10.1%)	2,568 (36.0%)	1,989 (5.6%)	1,027 (24.9%)	812 (3.9%)	874 (28.8%)	691 (4.6%)
**Prior convictions**	10,855 (11.2%)	4,924 (30.4%)	5,931 (7.3%)	770 (40.4 %)	1,225 (12.8%)	2,275 (31.9%)	2,441 (6.8%)	1,182 (28.7%)	1,487 (7.2%)	697 (23.0%)	778 (5.1%)

© 2026 Tayebi N et al. *JAMA Network Open*.

eTable 2. Descriptive statistics for the general population-matched sample without comorbid SUD.

	No. (%)										
	**Total sample *N*=474,294**			**Resource-limited neighborhoods *N*=41,106**		**Rural low-diversity neighborhoods *N*=219,876**		**Urban professional neighborhoods *N*=106,572**		**Urban affluent neighborhoods *N*=106,740**	
	Total	Cases *n*=79,049	Controls *n*=395,245	Cases, *n*=6,851	Controls *n*=34,255	Cases *n*=36,646	Controls *n*=183,230	Cases *n*=17,762	Controls *n*=88,810	Cases *n*=17,790	Controls *n*=88,950
Age at first diagnosis, y, median (IQR)	NA	20 (17-24)	NA	21 (18-25)	NA	19 (17-23)	NA	22 (19-26)	NA	18 (17-22)	NA
Sex											
Male	171,816 (36.2%)	28,636 (36.2%)	143,180 (36.2%)	2,478 (36.2%)	12,390 (36.2%)	13,487 (36.8%)	67,435 (36.8%)	6,209 (35.0%)	31,045 (35.0%)	6,462 (36.3%)	32,310 (36.3%)
Female	302,478 (63.8%)	50,413 (63.8%)	252,065 (63.8%)	4,373 (63.8%)	21,865 (63.8%)	23,159 (63.2%)	115,795 (63.2%)	11,553 (65.0%)	57,765 (65.0%)	11,328 (63.7%)	56,640 (63.7%)
Criminal convictions											
Violent	5,598 (1.2%)	1,334 (1.7%)	4,264 (1.1%)	159 (2.3 %)	764 (2.2%)	782 (2.1%)	2,187 (1.2%)	186 (1.0%)	689 (0.8%)	207 (1.2%)	624 (0.7%)
Non-violent	22,620 (4.8%)	4,738 (6.0%)	17,882 (4.5%)	501 (7.3%)	2,845 (8.3%)	2,648 (7.2%)	8,701 (4.7%)	773 (4.4%)	3,004 (3.4%)	816 (4.6%)	3,332 (3.7%)
Prior convictions	27,688 (5.8%)	5,579 (7.1%)	22,109 (5.6%)	714 (10.4 %)	3,658 (10.7%)	2,751 (7.5%)	9,576 (5.2%)	1,266 (7.1%)	5,414 (6.1%)	848 (4.8%)	3,461 (3.9%)

© 2026 Tayebi N et al. *JAMA Network Open*.

eTable 3. Association between depression and violent and non-violent criminal convictions, stratified by neighborhood type and comorbid SUD.

	With comorbid SUD (*N*=97,176)			Without comorbid SUD (*N*=474,294)		
	N	**Violent crime[a]**	**Non-violent crime[a]**	***N***	**Violent crime[a]**	**Non-violent crime[a]**
Resource-Limited Neighborhoods	11,442	5.49 (4.55–6.62)	6.50 (5.70–7.40)	41,106	1.04 (0.88–1.24)	0.86 (0.78–0.96)
Rural Low-Diversity Neighborhoods	42,786	10.34 (9.19–11.63)	11.42 (10.57–12.33)	219,876	1.82 (1.68–1.98)	1.59 (1.52–1.66)
Urban Professional Neighborhoods	24,738	10.77 (8.85–13.10)	9.18 (8.22–10.24)	106,572	1.36 (1.15–1.60)	1.31 (1.21–1.43)
Urban Affluent Neighborhoods	18,210	10.12 (8.12–12.60)	10.08 (8.90–11.42)	106,740	1.68 (1.43–1.97)	1.25 (1.15–1.35)

Analyses are based on the general population-matched sample. Data are odds ratios and 95% CIs.
[a]Data are unadjusted estimates.

© 2026 Tayebi N et al. *JAMA Network Open*.

eTable 4. Descriptive statistics for the general population-matched sample, excluding cases with depressive episodes with psychotic symptoms and their matched controls.

	No. (%)										
	**Total sample *N*=569,382**			**Resource-limited neighborhoods (*N*=52,356)**		**Rural low-diversity neighborhoods (*N*=261,576)**		**Urban professional neighborhoods (*N*=130,914)**		**Urban affluent neighborhoods (*N*=124,536)**	
	Total	Cases *n*=94,897	Controls *n*=474,485	Cases *n*=8,726	Controls *n*=43,630	Cases *n*=43,596	Controls *n*=217,980	Cases *n*=21,819	Controls *n*=109,095	Cases *n*=20,756	Controls *n*=103,780
Age at first diagnosis, y, median (IQR)	NA	20 (17-24)	NA	21 (18-25)	NA	19 (17-24)	NA	22 (19-26)	NA	18 (17-22)	NA
Sex											
Male	216,816 (38.1%)	36,136 (38.1%)	180,680 (38.1%)	3,360 (38.5%)	16,800 (38.5%)	16,859 (38.7%)	84,295 (38.7%)	8,055 (36.9%)	40,275 (36.9%)	7,862 (37.9%)	39,310 (37.9%)
Female	352,566 (61.9%)	58,761 (61.9%)	293,805 (61.9%)	5,366 (61.5%)	26,830 (61.5%)	26,737 (61.3%)	133,685 (61.3%)	13,764 (63.1%)	68,820 (63.1%)	12,894 (62.1%)	64,470 (62.1%)
Criminal convictions											
Violent	8,461 (1.5%)	3,064 (3.2%)	5,397 (1.1%)	413 (4.7 %)	1,047 (2.4%)	1,684 (3.9%)	2,724 (1.3%)	511 (2.3%)	865 (0.8%)	456 (2.2%)	761 (0.7%)
Non-violent	32,102 (5.6%)	9,875 (10.4%)	22,227 (4.7%)	1,204 (13.8%)	3,784 (8.7%)	5,193 (11.9%)	10,638 (4.9%)	1,789 (8.2%)	3,802 (3.5%)	1,689 (8.1%)	4,003 (3.9%)
Psychiatric disorders											
SUD	36,598 (6.4%)	16,148 (17.0%)	20,450 (4.3%)	1,903 (21.8%)	2,748 (6.3%)	7,108 (16.3%)	8,547 (3.9%)	4,110 (18.8%)	5,239 (4.8%)	3,027 (14.6%)	3,916 (3.8%)
ADHD	47,167 (8.3%)	25,794 (27.2%)	21,373 (4.5%)	2,506 (28.7%)	2,375 (5.4%)	12,597 (28.9%)	10,616 (4.9%)	5,087 (23.3%)	3,828 (3.5%)	5,604 (27.0%)	4,554 (4.4%)
Prior convictions	38,389 (6.7%)	10,463 (11.0%)	27,926 (5.9%)	1,480 (17.0 %)	4,858 (11.1%)	5,001 (11.5%)	11,961 (5.5%)	2,441 (11.2%)	6,881 (6.3%)	1,541 (7.4%)	4,226 (4.1%)

© 2026 Tayebi N et al. *JAMA Network Open*.

eTable 5. Associations between depression and violent and non-violent criminal convictions across neighborhood types, excluding cases with depressive episodes with psychotic symptoms and their matched controls.

	Violent			Non-violent	
	N	**Unadjusted OR**	**Adjusted OR[a]**	**Unadjusted OR**	**Adjusted OR[a]**
Resource-Limited Neighborhoods	52,356	2.08 (1.85–2.35)	1.13 (0.97–1.32)	1.76 (1.64–1.90)	1.02 (0.93–1.12)
Rural Low-Diversity Neighborhoods	261,576	3.27 (3.07–3.48)	1.51 (1.39–1.64)	2.76 (2.67–2.87)	1.50 (1.42–1.56)
Urban Professional Neighborhoods	130,914	3.07 (2.74–3.43)	1.48 (1.27–1.72)	2.59 (2.43–2.75)	1.35 (1.25–1.46)
Urban Affluent Neighborhoods	124,536	3.12 (2.77–3.51)	1.52 (1.30–1.78)	2.29 (2.16–2.44)	1.28 (1.19–1.39)

Analyses are based on the general population-matched sample. Data are odds ratios and 95% CIs.
[a]Adjusted for prior convictions, SUD, and ADHD.

© 2026 Tayebi N et al. *JAMA Network Open*.

eReferences

1. Ludvigsson JF, Otterblad-Olausson P, Pettersson BU, Ekbom A. The Swedish personal identity number: possibilities and pitfalls in healthcare and medical research. *Eur J Epidemiol.* 2009;24(11):659-667. doi:10.1007/s10654-009-9350-y
2. Ludvigsson JF, Almqvist C, Bonamy AKE, et al. Registers of the Swedish total population and their use in medical research. *Eur J Epidemiol.* 2016;31(2):125-136. doi:10.1007/s10654-016-0117-y
3. Ludvigsson JF, Andersson E, Ekbom A, et al. External review and validation of the Swedish national inpatient register. *BMC Public Health*. 2011;11(1):450. doi:10.1186/1471-2458-11-450
4. Ekbom A. The Swedish multi-generation register. In: Dillner J, ed. Methods in Biobanking. New York, NY: Humana Press; 2011:215-220. doi:10.1007/978-1-59745-423-0_10
5. Ludvigsson JF, Svedberg P, Olén O, Bruze G, Neovius M. The longitudinal integrated database for health insurance and labour market studies (LISA) and its use in medical research. *Eur J Epidemiol.* 2019;34(4):423-437. doi:10.1007/s10654-019-00511-8
6. Statistikmyndigheten SCB. DeSO – demografiska statistikområden. Accessed February 4, 2025. https://www.scb.se/hitta-statistik/regional-statistik-och-kartor/regionala-indelningar/deso---demografiska-statistikomraden/
7. Fazel S, Långström N, Hjern A, Grann M, Lichtenstein P. Schizophrenia, substance abuse, and violent crime. *JAMA*. 2009;301(19):2016-2023. doi:10.1001/jama.2009.675
8. Fovet T, Geoffroy PA, Vaiva G, Adins C, Thomas P, Amad A. Individuals with bipolar disorder and their relationship with the criminal justice system: a critical review. *Psychiatr Serv.* 2015;66(4):348-353. doi:10.1176/appi.ps.201400104
9. Everhov ÅH, Frisell T, Osooli M, et al. Diagnostic accuracy in the Swedish national patient register: a review including diagnoses in the outpatient register. *Eur J Epidemiol.* 2025;40(3):359-369. doi:10.1007/s10654-025-01221-0
10. Andersson A, Tayebi N, Isakovic B, et al. Neighborhood social structure in Sweden: A latent transition analysis using registry data from 1991 to 2020. *Cities.* 2026;168:106466. doi:/10.1016/j.cities.2025.106466
11. Moore KE, Oberleitner LMS, Zonana HV, et al. Psychiatric disorders and crime in the us population: results from the National Epidemiologic Survey on Alcohol and Related Conditions Wave III. *J Clin Psychiatry.* 2019;80(2):18m12317. doi:10.4088/JCP.18m12317
12. Virtanen S, Kuja-Halkola R, Mataix-Cols D, et al. Comorbidity of substance misuse with anxiety-related and depressive disorders: a genetically informative population study of 3 million individuals in Sweden. *Psychol Med.* 2020;50(10):1706-1715. doi:10.1017/S0033291719001788
13. Karriker-Jaffe KJ. Neighborhood socioeconomic status and substance use by U.S. adults. *Drug Alcohol Depend.* 2013;133(1):212-221. doi:10.1016/j.drugalcdep.2013.04.033
14. Ford JA, Sacra SA, Yohros A. Neighborhood characteristics and prescription drug misuse among adolescents: The importance of social disorganization and social capital. *Int J Drug Policy.* 2017;46:47-53. doi:10.1016/j.drugpo.2017.05.001
15. Schofield P, Thisted Horsdal H, Das-Munshi J, et al. A comparison of neighbourhood level variation and risk factors for affective versus non-affective psychosis. *Schizophr Res.* 2023;256:126-132. doi:10.1016/j.schres.2022.05.015
16. James SH, Galvan T, Zollicoffer A, Strauss GP. A meta-analysis of the role of neighborhood deprivation in psychotic disorders. *Soc Psychiatry Psychiatr Epidemiol.* 2025;60(12):2721-2733. doi:10.1007/s00127-025-02980-7

© 2026 Tayebi N et al. *JAMA Network Open*.
