# Supplementary material for: Depression and Crime Across Different Neighborhoods in the Swedish General Population
Source: JAMA Netw Open. 2026 Feb 3;9(2):e2557546. doi: 10.1001/jamanetworkopen.2025.57546 (PMC12869338; doi:10.1001/jamanetworkopen.2025.57546)
Supplement: Supplement 2. — Data Sharing Statement [file jamanetwopen-e2557546-s002.pdf]

# Data Sharing Statement

Tayebi. Depression and Crime Across Different Neighborhoods in the Swedish General Population. *JAMA Netw Open*. Published February 03, 2026.
doi:10.1001/jamanetworkopen.2025.57546

**Data**
**Data available:** No

**Additional Information**
**Explanation for why data not available:** Data cannot be shared publicly because of the Swedish Secrecy Act. Data from the Total Population Register, the National Patient Register, the National Crime Register, the Multi-Generation Register, the LISA register, and the DeSO Register were used for this study and made available by ethical approval. Researchers may apply for access through the Swedish Research Ethics Boards (www.etikprovningsmyndigheten.se) and from the primary data owners Statistics Sweden (www.scb.se) and the National Board of Health and Welfare (www.socialstyrelsen.se), in accordance with Swedish law.
